# Sensitive Detection of Hydroxytyrosol in Extra Virgin Olive Oils with a Novel Biosensor Based on Single-Walled Carbon Nanotubes and Tyrosinase

**DOI:** 10.3390/ijms23169132

**Published:** 2022-08-15

**Authors:** Alexandra Virginia Bounegru, Constantin Apetrei

**Affiliations:** Department of Chemistry, Physics and Environment, Faculty of Sciences and Environment, “Dunărea de Jos” University of Galaţi, 47 Domnească Street, 800008 Galaţi, Romania

**Keywords:** hydroxytyrosol, biosensor, tyrosinase, extra virgin olive oil, galvinoxyl

## Abstract

Hydroxytyrosol (HT) is an important marker for the authenticity and quality assessment of extra virgin olive oils (EVOO). The aim of the study was the qualitative and quantitative determination of hydroxytyrosol in commercial extra virgin olive oils of different origins and varieties using a newly developed biosensor based on a screen-printed electrode modified with single-layer carbon nanotubes and tyrosinase (SPE-SWCNT-Ty). The enzyme was immobilized on a carbon-based screen-printed electrode previously modified with single-layer carbon nanotubes (SPE-SWCNT-Ty) by the drop-and-dry method, followed by cross-linking with glutaraldehyde. The modified electrode surface was characterized by different methods, including electrochemical (cyclic voltammetry (CV), differential pulse voltammetry (DPV), electrochemical impedance spectroscopy (EIS)) and spectrometric (Fourier transform infrared (FTIR) spectroscopy) methods. Cyclic voltammetry was used for the quantitative determination of HT, obtaining a detection limit of 3.49 × 10^−8^ M and a quantification limit of 1.0 × 10^−7^ M, with a wide linearity range (0.49–15.602 µM). The electrochemical performance of the SPE-SWCNT-Ty biosensor was compared with that of the modified SPE-SWCNT sensor, and the results showed increased selectivity and sensitivity of the biosensor due to the electrocatalytic activity of tyrosinase. The results obtained from the quantitative determination of HT showed that commercial EVOOs contain significant amounts of HT, proving the high quality of the finished products. The determination of the antiradical activity of HT was carried out spectrophotometrically using the free reagent galvinoxyl. The results showed that there is a very good correlation between the antiradical capacity of EVOOs, the voltammetric response and implicitly the increased concentration of HT. SPE-SWCNT-Ty has multiple advantages such as sensitivity, selectivity, feasibility and low cost and could be used in routine analysis for quality control of food products such as vegetable oils.

## 1. Introduction

*Olea europaea* L., commonly known as the olive tree, is a small tree species found mostly in Mediterranean countries, with olive oil as its main by-product [1]. Olive oil is present in the Mediterranean diet, and it is considered a protective factor in the prevention of heart disease. Research has shown that the health benefits of the Mediterranean diet are largely attributed to olive oil precisely because of its antioxidant [2,3] anti-tumour [4,5] anti-inflammatory [6], hypolipidemic [7] and even antimicrobial [8,9] action.

Virgin olive oil (VOO) is obtained by extraction, first crushing the olive fruit in a hammer mill to form a paste. The olive oil is then separated and centrifuged for clarification and purification [10].

In terms of composition, VOO contains mainly triglycerides (97–99%) and an unsaponifiable fraction (1–3%) containing mostly phenolic compounds responsible for its biological properties but also organoleptic attributes [11,12].

Oleuropein, hydroxytyrosol and tyrosol make up the majority of the phenolic fraction in olive oil [13,14]. In addition to these, fat-soluble vitamins such as tocopherols, hydrocarbons (squalene) or pigments such as chlorophyll and carotenoids can be identified [15,16].

The main group of antioxidants in VOO are hydrophilic phenols, compounds that are highly relevant in determining the quality of the oil in terms of the degree of bitter or spicy taste as well as stability [17,18] but also for the specific flavour of each VOO [19]. Each VOO has a different composition and phenolic content profile, which explains the different quality and stability characteristics [20].

Over 30 different phenolic compounds have been identified in VOO [21,22], including phenolic acids such as hydroxybenzoic, p-coumaric, ferulic, gallic, syringic, vanillic, caffeic, o-coumaric acids, and synaptic acids [19]. Other types of polyphenols that can also be found in VOO are flavonoids, lignans, hydroxyisochromans, secoiridoids and phenolic alcohols [23].

Secoiridoids are phenolic compounds with a small percentage in the composition of VOO, being insoluble in oil, and most of them are destroyed by the mechanical extraction process. However, they manage to imprint important biological and organoleptic properties on VOO [17]. The most common secoiridoids are demethyloleuropein, oleuropein, ligstrozide and their aglycones, with the latter accounting for about 90% of the phenolic compounds in VOO [23]. The bitter taste of VOO is due to the secoiridoids present, in particular the dialdehyde form of oleuropein aglicon [24].

Representatives of another class of compounds, the phenolic alcohols, present in VOO are tyrosol (p-Hydroxyphenylethanol) and hydroxytyrosol (2-[3,4-dihydroxyphenyl] ethanol). They are present in low concentrations in fresh olive oil but tend to increase during the storage process due to the hydrolysis of secoiridoids [25].

Among all the constituent compounds of VOO, oleuropein, hydroxytyrosol and tyrosol having an o-diphenolic structure are considered important markers for authenticity or correct preservation of olive oil.

Studies also show that the presence of tyrosol and hydroxytyrosol has an osteoprotective effect by stimulating calcium absorption [26] and proliferation of osteoblast cells [27], and oleuropein has a hypoglycemic [28], anti-tumour [29] and neuroprotective [30] effect.

The concentrations of these phenolic compounds depend on many aspects, such as climate, geographical origin, ripeness of the olive fruit at harvest, production conditions, storage conditions and storage time [31,32,33,34,35,36]. Precisely in order to ensure that VOO is of high quality and to avoid adulteration, the European Union Commission, the International Olive Oil Council, and the Codex Alimentarius Committee regulate and carefully monitor the content of certain phenolic compounds, such as oleuropein or hydroxytyrosol, which are considered specific chemical markers [37,38].

The classical analytical methods often used to evaluate these parameters are chromatography or spectrophotometry. They are efficient but involve long times, expensive reagents and additional sample preparation steps [39]. Alternative methods include electrochemical techniques that provide fast and accurate results [40]. Electrochemical sensors and biosensors have become increasingly diverse both structurally and in the functionalisation of the nanomaterials used as carriers to make determinations as sensitive, selective and easy as possible, using small amounts of samples without the need for complex pre-preparation [41,42,43,44,45,46]. The literature provides valuable information on sensitive and selective techniques, sensors and biosensors for the determination of oleuropein, tyrosol and hydroxytyrosol from real samples of VOO [47,48,49,50]. In most papers, the electrochemical method (e.g., DPV, CV) is combined with liquid-liquid extractive methods and compared with spectrophotometric or chromatographic methods [51,52,53]. In the case of biosensors, the most frequently immobilised were tyrosinase [54,55,56] and peroxidase [57] enzymes capable of catalysing the oxidation of monophenols to form o-phenols, which in turn are oxidised to o-quinones. Using an electrochemical technique, the quinones are reduced, and the measured current is proportional to the concentration of the phenolic compound in the VOO [37,58].

The objective of the present paper is to construct a reliable, sensitive and selective tyrosinase-based biosensor for hydroxytyrosol determination and quantification from real olive oil samples. Tyrosinase (Ty) will be immobilized by casting and cross-linking after prior modification of a carbon screen-printed electrode with single-walled carbon nanotubes. This immobilization method was chosen because the reduction of quinones on the carbon nanomaterial particles generates o-diphenols which will in turn be reoxidized by enzymes located around the carbon particles, thus amplifying the electrochemical process [59]. Characterisation of the biosensor surface will be performed by both electrochemical (CV, DPV, EIS) and spectrometric (FTIR) methods. Calibration curves for HT will be performed by cyclic voltammetry (CV). Quantitative determinations will be made from different real olive oil samples using CV. In parallel, real samples will also be analysed spectrophotometrically, determining the antiradical capacity which will be correlated with the voltammetric data.

## 2. Results and Discussion

### 2.1. Surface Characterisation of Electrodes

In the first stage of the study, the surface characterization of the electrodes is performed by cyclic voltammetry, not before optimizing the potential range. Both modified electrodes (SPE-SWCNT and SPE-SWCNT-Ty) gave a stable signal in the potential range −0.4 and +1.3 V. Therefore, it was maintained for all determinations in active solutions and real samples.

**Figure 1:**
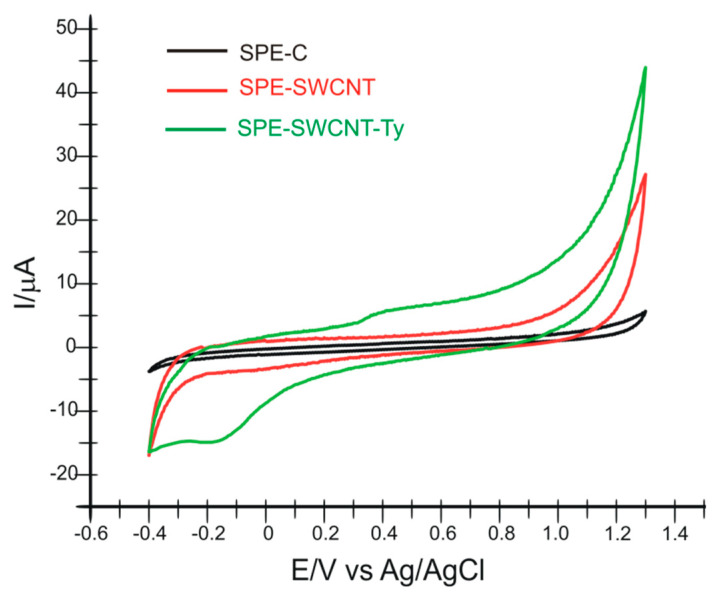
Cyclic voltammograms recorded by the three sensors in 10^−1^ M PBS solution, pH 7.0, scan rate 0.1 V·s^−1^.

Upon immersion of the three modified sensors in 10^−1^ M PBS electrolyte solution (pH 7.0), cyclic voltammograms were recorded, showing the presence of single-layer carbon nanotubes in the case of SPE-SWCNT and tyrosinase in the case of the SPE-SWCNT-Ty biosensor. The scan rate was 0.1 V·s^−1^. The oxidation and reduction of tyrosinase can be observed at *E*_pa_ = 0.400 V (*I*_pa_ = 5.762 μA) and *E*_pc_ = −0.165 V (*I*_pc_ = −14.514 μA), respectively. Figure 1 shows the cyclic voltammograms recorded by the three sensors in PBS solution 10^−1^ M, pH 7.0.

**Figure 2:**
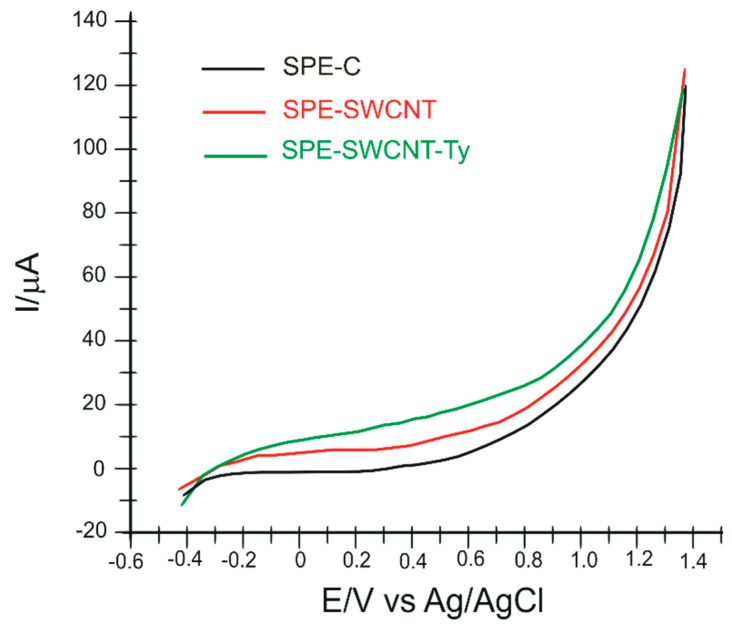
Differential pulse voltammograms recorded by the three sensors in 10^−1^ M PBS solution, pH 7.0, scan rate 0.1 V·s^−1^.

The second stage of sensor characterization was performed using differential pulse voltammetry. Thus, the three electrodes were immersed in 10^−1^ M PBS electrolyte solution (pH 7.0), and the recorded voltammograms can be seen in Figure 2. The optimized parameters were: pulse height, 7.0 mV; pulse width, 100 ms; scan rate, 0.1 V·s^−1^; and potential range −0.4 and +1.3 V. From the recorded voltammograms, a significant difference can be observed in the biosensor, which reveals the presence of immobilized tyrosinase on the electrode surface. The oxidation of the enzyme is noted by the appearance of a faint but obvious peak compared to the other two sensors, at *I*_pa_ = 17.859 μA and *E*_pa_ = 0.563 V. The potential was measured where the greatest difference in current intensity was noted between the voltammograms of the three electrodes.

The next step was to investigate the electrode surface by electrochemical impedance spectroscopy (EIS).

EIS is a technique by which the electrical properties of a wide variety of materials can be investigated. In EIS, a sinusoidal test voltage or current is applied to a sample to measure its impedance over a suitable frequency range. In practice, the measured impedance spectra represent an electrical fingerprint of the sample, providing valuable information on its properties and behaviour [60]. Through EIS, the electron transfer properties of the surface of the modified electrodes can be characterised. The semicircle diameters of the Nyquist diagram reflect the electron transfer resistance (*R*_ct_), and the linear part corresponds to the Warburg diffusion process. In EIS, the Radles circuit is most often used. This is an equivalent electrical circuit in which all the current passes through the solution, which acts as an Ohmic resistor *R*_s_ [61]. Thus, the circuit comprises a solution resistance (*R*_s_), a charge transfer resistance *R*_ct_), a double layer capacitance (*C*_dl_) and Warburg impedance (*Z_W_*) [62].

**Figure 3:**
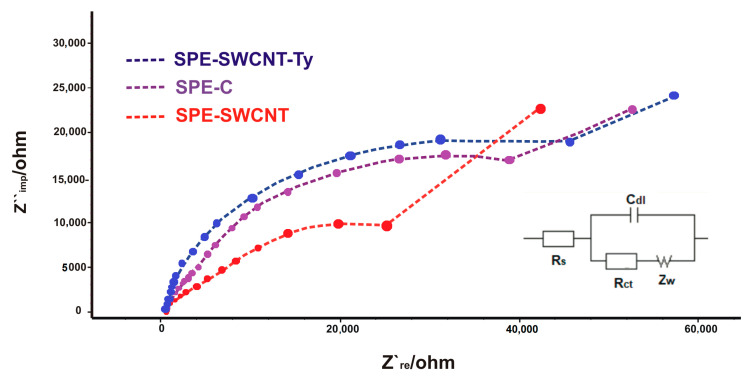
Nyquist plots of EIS for SPE-SWCNT (red line), SPE-C (purple line), SPE-SWCNT-Ty (blue line) in 10^−1^ M KCl and 10^−3^ M [Fe(CN)_6_]^3−/4−^ for a frequency range of 0.01 Hz to 10 kHz, amplitude 10 mV. Inset: Equivalent circuit applied to fit the impedance spectra.

Electrodes were immersed and subjected to EIS analysis in an electrochemical cell containing K_3_ [Fe(CN)_6_]/K_4_ [Fe(CN)_6_] 10^−3^ M and KCl 10^−1^ M in a 1:1 ratio (Figure 3).

Figure 3 shows Nyquist plots of the impedance spectroscopy of the three electrodes. The *R*_ct_ values increased in order: SPE-SWCNT < SPE-C < SPE-SWCNT-Ty. The *R*_ct_ value of SPE-SWCNT (25,129.4 Ω) was lower than that of SPE-C (39,615.4 Ω), demonstrating that the modification with single-walled carbon nanotubes allowed a higher penetration of the Fe(CN)_6_^3−/4−^ redox sample than in the case of carbon; therefore, it improves electron transfer between the analyte and the electrode surface. An obvious increase in resistance was observed when Ty was immobilized on the SPE-SWCNT (*R*_ct_ = 52,992.6 Ω) surface, as the macromolecular structure of tyrosinase prevents electron transfer. This electrochemical behaviour of the tyrosinase-based biosensor is also consistent with other studies [63,64].

In order to observe the changes in the modified carbon-based sensor and biosensor, the active surface of the three working electrodes was analysed with the FTIR method.

**Figure 4:**
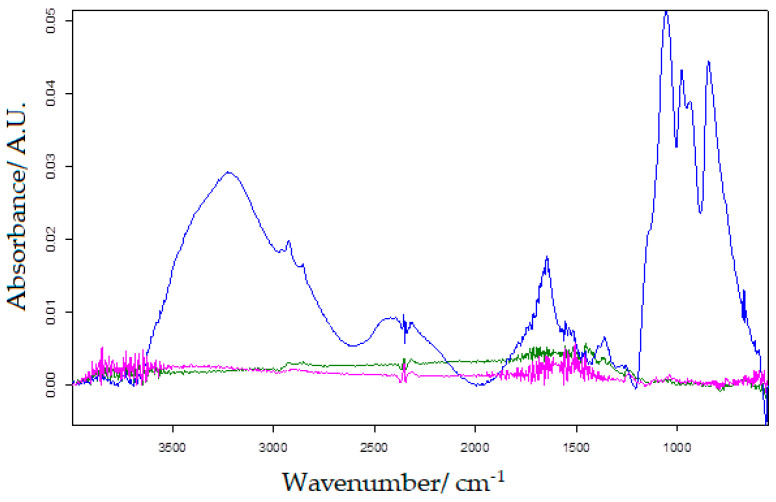
FTIR for SPE-C (pink line), SWCNT-SPE (green line) and SPE-SWCNT-Ty (blue line).

Figure 4 shows the FTIR spectra for SPE-C, MWCNT-SPE and MWCNT-Ty-SPE, respectively, and the differences in peak number and background noise are evident. Several peaks representing the presence of tyrosinase can be observed in the wavenumber range 3500–2800 cm^−1^ and 1200–500 cm^−1^ [65].

### 2.2. The Voltammetric Responses of Biosensor in Hydroxytyrosol Solution

**Figure 5:**
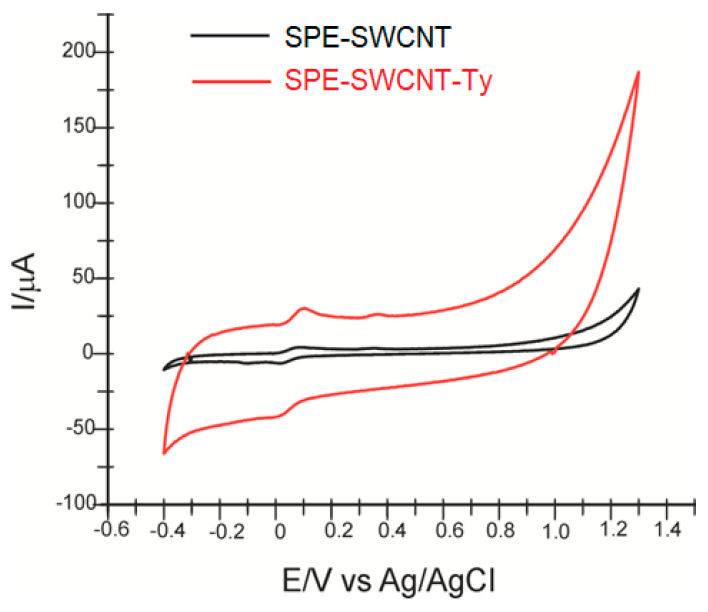
Cyclic voltammograms of SPE-SWCNT (black line) and SPE-SWCNT-Ty (red line) in PBS solution 10^−1^ M (pH 7.0) containing 10^−4^ M HT. Scan rate: 0.1 V·s^−1^.

The two newly constructed electrodes, SPE-SWCNT and SPE-SWCNT-Ty, were used for hydroxytyrosol detection studies with higher sensitivity and better selectivity. Figure 5 shows the cyclic voltammograms of the two electrodes in hydroxytyrosol (HT) 10^−4^ M—PBS 10^−1^ M solution (pH = 7.0).

In both cases, two anodic and one cathodic peak of different intensities and potentials are shown, related to the oxidation or reduction of hydroxytyrosol at the sensitive element, respectively. The cyclic voltammograms show slightly different results depending on the changes to the working electrode used. This electrochemical behaviour is similar to that observed in other previously published studies [66]. Table 1 shows the results obtained from the redox peak pair analysis observed in the cyclic voltammograms.

In the case of SPE-SWCNT-Ty, the cathodic peak potential has a lower value, with this shift towards negative potential values indicating that the reduction process is strongly influenced by the presence of the enzyme [67,68]. The detection at a lower potential indicates that the reduction process requires a lower activation energy in the case of the biosensor [69].

**Figure 6:**
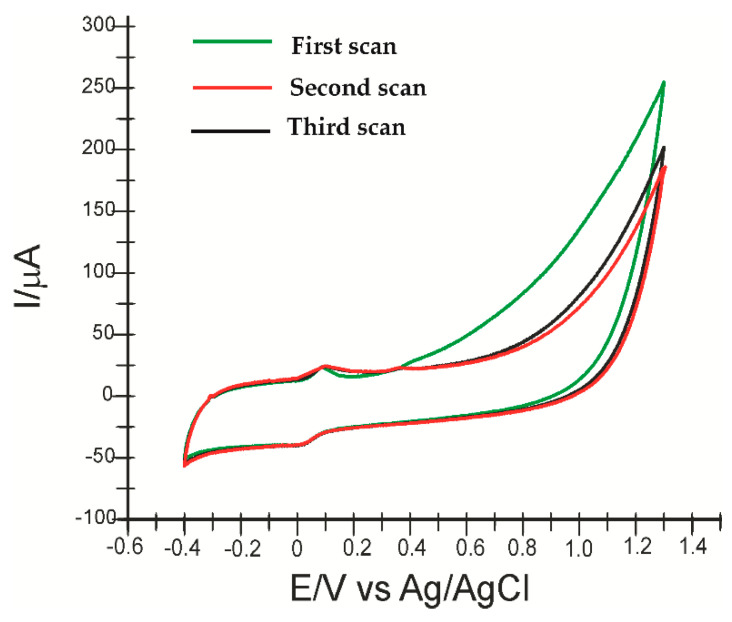
Cyclic voltammogram of SPE-SWCNT-Ty immersed in 10^−4^ M HT acid solution (electrolyte PBS 10^−1^ M, pH = 7.0, scan rate 0.1 V·s^−1^): first scan (green line), second scan (red line) and third scan (black line).

The electrooxidation and electroreduction reactions of HT for the biosensor performed in this study are shown in Figure 6 (for 3 successive scans).

**Figure 7:**
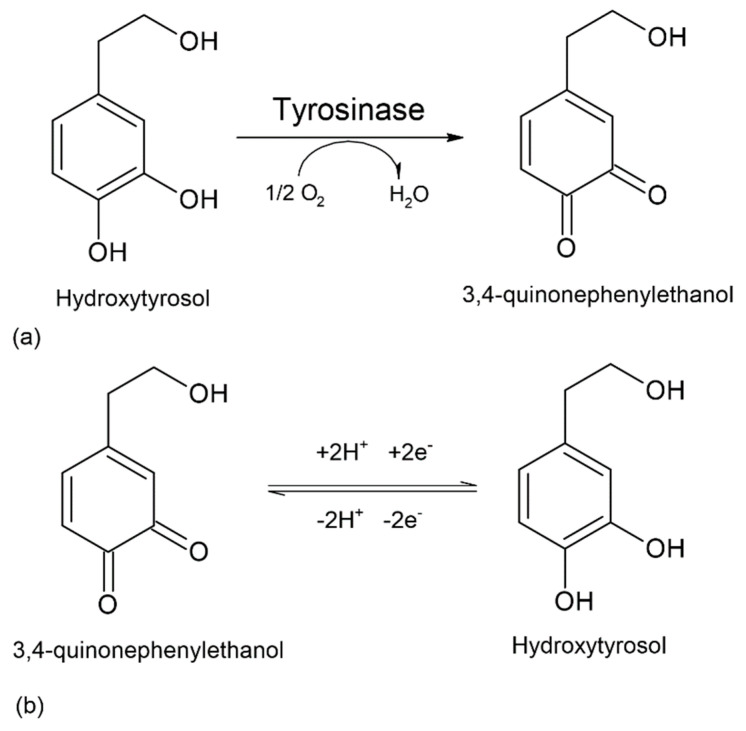
(**a**) Tyrosinase-catalysed oxidation of hydroxytyrosol; (**b**) reversible electrochemical reduction of hydroxytyrosol at the biosensor surface.

The potential range was maintained according to the first experiments, and the scan rate used was 0.1 V·s^−1^. At the first voltametric scan, a weakly evidenced, irreversible anodic peak appears, which is associated with the oxidation of hydroxyl groups on the aromatic ring of the molecule and the formation of the corresponding ortho-quinone (3,4-quinophenylethanol), a reaction catalysed by tyrosinase (diphenolase activity) [70]. In the case of the biosensor, the anodic peak occurs at a potential of 0.369 V. The oxidation reaction of hydroxytyrosol catalysed by tyrosinase is shown in Figure 7.

On successive scans, the oxidation product of HT, being very unstable, accumulates on the electrode surface, forming a polymer film, which explains the appearance of another reversible oxidation peak, well evidenced at a lower potential [71].

Additionally, the low value of *E*_pc_ suggests a rapid electron transfer process occurring at the active surface of the biosensor in the case of oxidation-reduction of HT [72].

Therefore, SPE-SWCNT-Ty shows better selectivity compared to SPE-SWCNT in HT detection, thus confirming the biocatalytic activity of tyrosinase immobilized on the biosensor surface. The values of the parameters *I*_pc_ and *I*_pa_ prove that the biosensor shows better sensitivity than the sensor.

The presence of tyrosinase predominantly influences the HT reduction process, which is confirmed by a higher intensity of the cathodic peak, which is why subsequent calculations will refer to its changes. In the case of SPE-SWCNT-Ty, the signal was more stable and the background noise lower.

**Figure 8:**
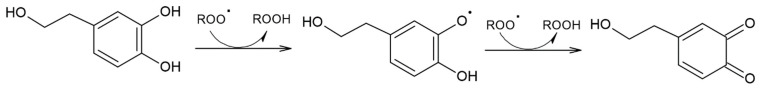
Free radical scavenging mechanism exerted by hydroxytyrosol (HT).

Moreover, HT also has strong antioxidant activity due to its high capacity to limit both intracellular and extracellular ROS production, being mainly effective with free molecules or radicals, such as H_2_O_2_ and O_2_, acting also as a metal chelator. These properties are due both to the presence of hydroxyl (OH) groups in the ortho position, which have an electron-donating capacity, and to HT’s ability to bind phenoxyl radicals, forming stable hydrogen bonds (Figure 8) [73].

### 2.3. Influence of Scanning Rate on the Voltammetric Response

**Figure 9:**
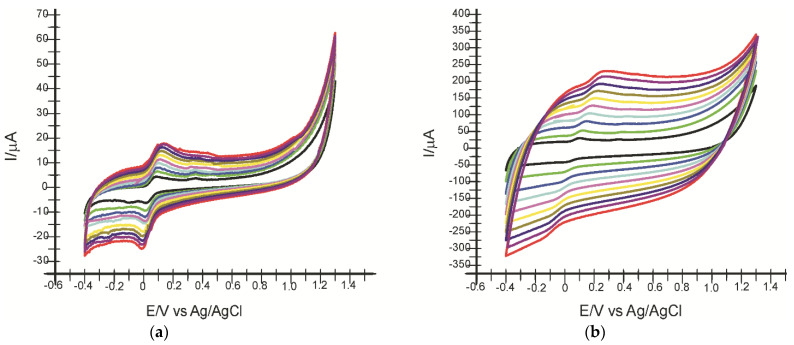
Cyclic voltammograms of SPE-SWCNT (**a**) and SPE-SWCNT-Ty (**b**) recorded at different scan rates in the range of 0.1–1.0 V·s^−1^. The colour correspond to different scan rates.

In the next step, the electrochemical behaviour of the two electrodes in HT 10^−4^ M solution was studied by applying increasing scanning rates in the range of 0.1–1.0 V·s^−1^. Remarkable differences between the intensities of the oxidation and reduction currents and the measured potentials are observed as early as the second scan rate applied, with the peaks progressively increasing with increasing scan rate. Since enzyme immobilization predominantly influences the cathodic peak, the dependence of *I*_pc_ on scan rate will be studied. Figure 9 shows the cyclic voltammograms of SPE-SWCNT and SPE-SWCNT-Ty recorded at different scan rates.

It was determined that there is a linear dependence between cathode peak currents and scanning rate for both electrodes (Table 1). This indicates that the process occurring at the electrode surface is controlled by the adsorption of the electroactive species, with HT adsorption on the active surface being the determining step in the kinetics of the electrochemical process [71].

Given the initial dependence equation between cathode peak current and scan rate, the degree of electrode surface coverage with the electroactive species (Γ) was calculated using the Laviron equation, and the results are shown in Table 2 [41].

(1) $$I_{\mathrm{pc}}=\frac{n^{2}F^{2}\Gamma \mathrm{Av}}{4\mathrm{RT}}$$

Comparing the results obtained with the two electrodes, it can be stated that in all cases, the reduction process is controlled by the HT adsorption on the active surface, which is faster and more evident in the case of the biosensor.

Table 2 shows the equations of the *I*_pc_ vs. dependencies, the coefficients of determination (R^2^) and the degree of electrode surface coverage with the electroactive species (Γ).

From these results, it can be appreciated that SPE-SWCNT-Ty has superior electroanalytical properties for HT detection. In addition, the presence of tyrosinase ensures the selectivity of the biosensor and can be used in the analysis of complex samples. Immobilization leads to better bioselectivity and conductivity. Since SPE-SWCNT-Ty has shown superior performance on sensitivity and selectivity, it will be used in further quantitative analyses.

### 2.4. Calibration Curve

From the experimental data previously obtained, it can be seen that the biosensor shows superior performance to the sensor due to the presence of the enzyme that gives it selectivity and sensitivity and favours the interaction with hydroxytyrosol; therefore, for the determination of the calibration curve, cyclic voltammograms will be recorded only with SPE-SWCNT-Ty.

For this step, varying amounts between 5 and 50 μL of HT 10^−4^ M stock solution were successively added to 50 mL PBS solution 10^−1^ M pH 7.0 under continuous stirring. The concentration range studied was 0.01–28.62 μM.

**Figure 10:**
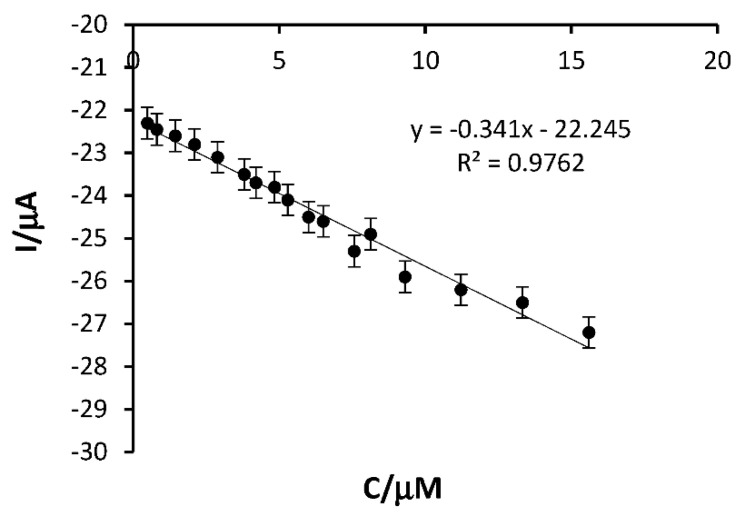
Linear fit of SPE-SWCNT-Ty response in the concentration range 0.49–15.602 μM.

As can be seen in Figure 10, the cathodic peak current increases with increasing HT concentration, demonstrating the stable and efficient catalytic capacity of the biosensor. The response current was linear in the range of 0.49–15.602 μM.

Using the linear regression equation, LOD (3σ/m, where σ was the standard deviation and m was the slope of the calibration curve) and LOQ (10σ/s) [74] were calculated, and the values are shown in Table 3.

The newly developed biosensor is found to exhibit detection and quantification limits in the nanomolar range, demonstrating increased sensitivity for HT detection.

Using the calibration curve values, *I*_max_ was determined and then graphically represented as log(*I*/(*I*_max_ − *I*)) vs. log(HT). From the line equation, the Hill coefficient (*h*) was extracted and represented by the slope of the line. SPE-SWCNT-Ty showed a Hill coefficient with a value near 1, which means that the overall process at the biosensor surface exhibits Michaelis–Menten kinetics. An obtained value of h lower than 1 reflects a negative cooperative effect between occupied active zones on the SPE-SWCNT-Ty surface. Next, the Lineweaver–Burk equation was used to calculate the Michaelis–Menten constant.

(2) $$\frac{1}{I}=\frac{1}{I_{\max}}+\frac{K_{M}^{app}}{I_{\max}\left[\mathrm{HT}\right]}$$

where *I* is the cathode current, *I*_max_ is the steady state current, $K_{M}^{app}$ is the apparent Michaelis–Menten constant, and [HT] is the substrate concentration. From the ordinate of the origin, the value of *I*_max_ is calculated, and from the slope of the line, the value of $K_{M}^{app}$ is calculated. The characteristic parameters of SPE-SWCNT-Ty for HT are given in Table 4.

The small value of the Michaelis–Menten constant indicates that the affinity between tyrosinase and hydroxytyrosol is strong for SPE-SWCNT-Ty.

### 2.5. Stability, Reproducibility, Repeatability and Interference Studies

**Figure 11:**
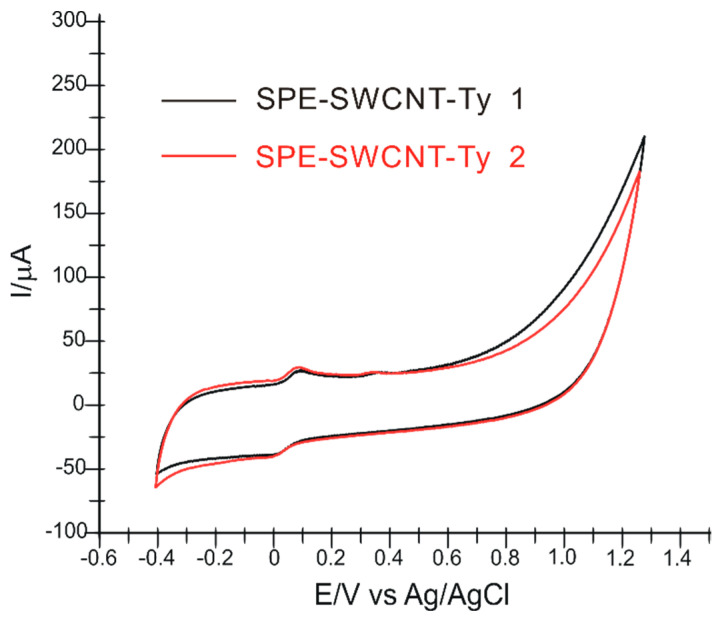
CVs recorded by two biosensors prepared identically (almost simultaneously) immersed in a solution of HT 10^−4^ M, scan rate 0.1 V × s^−1^.

The stability of the biosensor was studied, and it was found that it can be used for more than 30 measurements by cyclic voltammetry in solutions containing HT. Additionally, to check the reproducibility of the fabrication method, we studied the response of two identically prepared biosensors in HT solutions of concentrations of 10^−4^ M. There were no differences greater than 3% between the two biosensors (Figure 11).

To analyse the variation of the biosensor response to HT determination in solutions of the same concentration, the same biosensor was used but not before being removed from the solution and rinsed. When repeating the cyclic voltammogram, the difference was not more than 2.5%. Since the biosensor constructed is disposable, the result is very good.

For interference studies, the biosensor behaviour was evaluated at additions of compounds often found in olive oil, e.g., oleuropein and tyrosol. SPE-SWCNT-Ty showed good selectivity, with the potential and cathode peak current having insubstantial changes.

The HT solution had a concentration of 10^−4^ M, adding the same concentration of interferents.

The results are shown in Table 5.

Recovery (%) represents the percentage of the ratio between the current recorded after adding the interfering compounds and the current recorded with the biosensor in the absence of the interfering compounds. As can be seen in Table 5, the determination of HT is not significantly influenced by the interfering compounds studied, the error being 3.4% for oleuropein and 2.5% for tyrosol.

### 2.6. Determination of HT in Extra Virgin Olive Oils

The extra virgin olive oils (EVOO) selected for analysis were purchased from grocery stores (supermarkets) and are of different origins and varieties (Tunisia, Greece, Italy, and Spain). The samples to be analysed (12 commercial EVOO) were prepared in advance by extracting oil samples (5 g) using a 4:1 methanol:ultrapure water mixture. Samples were centrifuged, and the supernatant was recovered using a pipette and added to the electrochemical cell in 50 mL PBS, pH = 7.0.

**Figure 12:**
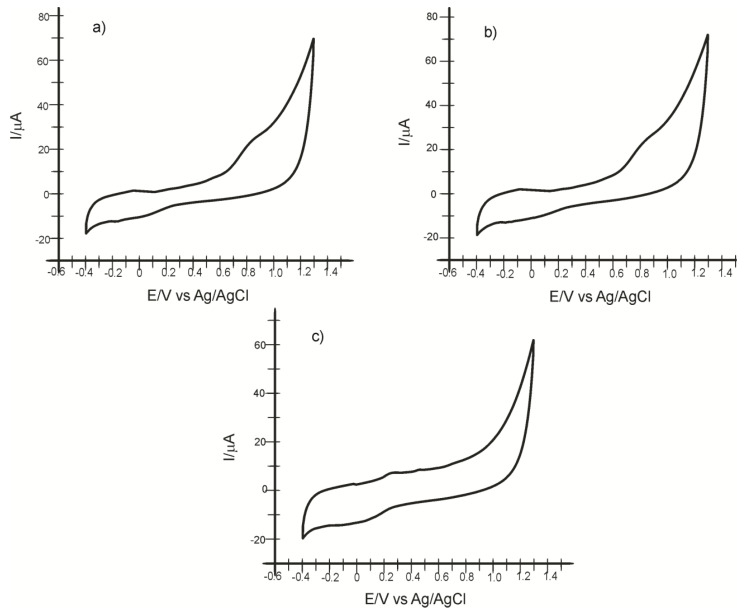
Cyclic voltammograms of the SPE-SWCNT-Ty biosensor immersed in samples of (**a**) Minerva Greek Extra Virgin Olive Oil, (**b**) Terra Delyssa Huile D”Olive De Tunisie, and (**c**) Monini Classico Olio Extra Vergine Di Oliva at a scan rate of 0.1 V×s^−1^.

Figure 12 shows the cyclic voltammograms of SPE-SWCNT-Ty immersed in solutions obtained in three of the EVOOs selected for analysis. Cyclic voltammograms recorded with SPE-SWCNT-Ty show peaks corresponding to the presence of HT in all samples to be analysed.

For quantification, the cathode peak intensity, corresponding to the potential −0.003 V, was used for each product. The results are included in Table 6.

The results obtained show a similar amount of HT present in the three commercial EVOOs. It is found that the Italian product Pietro Coricelli Olio Extra Vergine di Oliva presents a slightly higher amount of HT, which could make it superior both in taste, aroma and stability. According to the European Food Safety Authority, an amount of 5 mg of hydroxytyrosol and/or its derivatives should be consumed daily for the lipid-lowering and protective effect on the cardiovascular system [75]. Analyzing the obtained results, we can state that daily consumption of approximately 35 g of Pietro Coricelli Olio Extra Vergine di Oliva could cover the daily requirement of HT, which would be consistent with the amount of daily VOO recommended in other specialist studies [76].

### 2.7. Determination of the Antiradical Activity of Extra Virgin Olive Oils

To determine the antiradical activity, the stable radical galvinoxyl (O-centered radical) was used, being better associated with the physiological action of oxygen radicals and more sensitive to phenolic compounds than DPPH [77].

Galvinoxyl is reduced by scavenging hydrogen donor free radicals as shown in reaction (3) [78]:

G· + IH → GH + I·(3)

where G· is galvinoxyl; GH is reduced galvinoxyl; IH is a hydrogen donor free radical scavenger, in this case, HT; and I· is the corresponding radical of IH.

For analysis of the real samples, 300 μL extract was mixed with 2.9 mL free galvinoxyl radical solution. The radical-scavenging capacity (%RSC) was expressed as a percentage and was calculated using the following formula (4) [79]:

% RSC = (A_control_ − A_sample_)/A_control_ × 100 (4)

where A_control_ and A_sampl_ are the absorbances of the control and of the samples at 860 nm.

The %RSC results are included in Table 7. 

**Figure 13:**
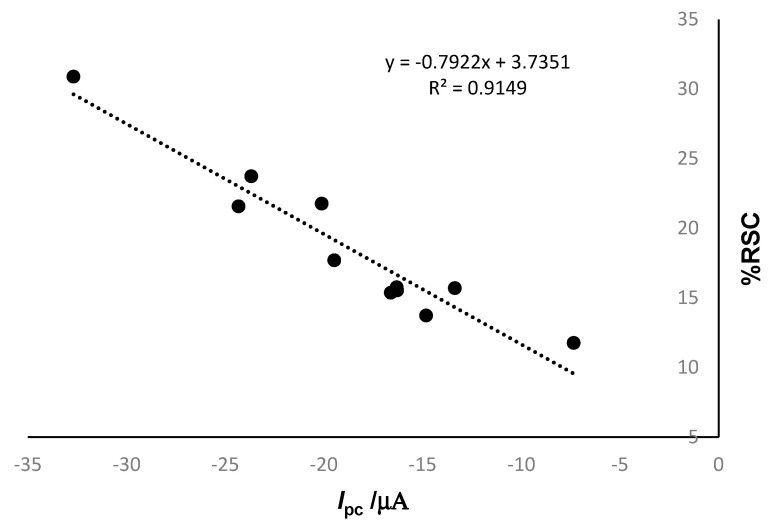
Correlation between RSC% and cathodic current related to HT reduction.

As can be seen in the table, the higher antiradical capacity is present in the product Pietro Coricelli Olio Extra Vergine di Oliva. This result can be correlated with a higher intensity of the cathodic peak and a higher HT content, as shown in Figure 13.

As can be observed in Figure 13, it is a good correlation between % RSC and the cathodic current corresponding to HT. This correlation could be useful in the estimation of RSC from the electrochemical data obtained with the biosensors by cyclic voltammetry.

## 3. Materials and Methods

### 3.1. Reagents and Samples

A screen-printed carbon electrode (SPE) purchased from Metrohm DropSens (Oviedo, Spain) was used to modify the sensors. The SPE was modified in the first step with a suspension prepared from single-walled carbon nanotube powder (Sigma-Aldrich, St Louis, MO, USA) dispersed in a mixture of dimethylformamide (DMF) (Sigma-Aldrich, St Louis, MO, USA) and ultrapure water (obtained with a Milli-Q system—Millipore, Bedford, MA, USA), yielding SPE-SWCNT which was subsequently used to construct the tyrosinase (Ty)-based biosensor.

Phosphate buffer solution 10^−1^ M (PBS), used as the supporting electrolyte in the electrochemical measurements, was prepared from NaH_2_PO_4_ and Na_2_HPO_4_, reagents purchased from Sigma-Aldrich, St Louis, MO, USA. pH was adjusted to 7.0 with the help of a pH meter (WTW, Weilheim, Germany). Potassium chloride, potassium ferro- and ferric cyanide (Sigma-Aldrich, St Louis, MO, USA) were used to prepare the solution used for electrochemical characterization of the electrodes by EIS.

Analytically pure hydroxytyrosol was purchased from Sigma-Aldrich, St Louis, MO, USA. For the preparation of the hydroxytyrosol stock solution (10^−4^ M), the appropriate amounts of the substances were dissolved in PBS solution at pH 7.0. Lyophilized tyrosinase powder (T3824-25KU, from mushroom) with a concentration of 8503 U/mg was purchased from Sigma-Aldrich. For the immobilization of the enzyme, tyrosinase solution of concentration 80 μg/µL dissolved in PBS buffer solution of pH 7.0 was used.

The compounds used for the interference studies (oleuropein and tyrosol) were purchased from Sigma-Aldrich.

Samples of virgin olive oils were prepared for analysis using liquid-liquid extraction [80]. An amount of 5 g of each oil was dissolved in a methanol-water mixture (40:10, *v*/*v*). The methanolic extracts were centrifuged, after which the supernatant was added to 50 mL PBS solution 10^−1^ M, pH 7.0. Methanol was purchased from Merck (Darmstadt, Germany).

The free galvinoxyl (2,6-di-tert-butyl-α-(3,5-di-tert-butyl-4-oxo-2,5–cyclohexadiene-1-ylidene)-p-tolyloxy) reagent was purchased from Sigma-Aldrich, St. Louis, MO, USA. The ethanolic solution of galvinoxyl radical was of concentration 1 mM. For the measurement of galvinoxyl radical scavenging activity, samples were prepared using a volume of 2.9 mL previously prepared free galvinoxyl reagent solution and 300 μL extract. For each prepared sample, the absorbance was measured relative to the control sample at wavelength 860 nm [81,82].

### 3.2. Equipment

#### 3.2.1. Electrochemistry

Electrochemical measurements were performed in an electrochemical cell (50 mL) with three electrodes, an Ag/AgCl reference electrode (Princeton, Applied Research, Princeton, NJ, USA), an auxiliary electrode represented by a platinum wire and a screen-printed working electrode (DropSens, Oviedo, Spain). The modified SPE-SWCNT screen-printed sensor was used as a working electrode first, and then the SPE-SWCNT-Ty biosensor. An Elmasonic ultrasonic bath (Carl Roth GmbH, Karlsruhe, Germany) was used for rapid dissolution of substances and homogenization.

Cyclic voltammetry (CV) and differential pulse voltammetry (DPV) measurements were performed with an EG&G potentiostat/galvanostat, model 263 A (Princeton Applied Research, Oak Ridge, TN, USA) controlled via Echem Software.

Surface characterization measurements of the modified electrode surfaces were performed by electrochemical impedance spectroscopy (EIS) using an SP 150 potentiostat/galvanostat controlled by EC-Lab Express software, to which an electrochemical cell (50 mL) with these three electrodes (reference electrode, auxiliary electrode and working electrode) was connected.

#### 3.2.2. Spectrophotometry

The galvinoxyl radical scavenging activity of each real sample extract was evaluated using the Rayleigh UV2601 UV/Vis double beam spectrophotometer (Beijing Beifen-Ruili Analytical Instrument, Beijing, China) at 860 nm wavelength. The instrument is controlled by UVSoftware.

#### 3.2.3. Spectrometry

A Bruker ALPHA-E FTIR spectrometer (BrukerOptik GmbH, Ettlingen, Germany) connected to OPUS software (BrukerOptik GmbH, Ettlingen, Germany) was used for the infrared spectrometric method. The FTIR spectrometric method was used to characterize the active surface of modified electrodes. The spectra were recorded in the range of 4000–500 cm^−1^ with 32 scans and a resolution of 4 cm^−1^ in attenuated total reflectance (ATR) mode. The ATR ZnSe crystal was rinsed with ultrapure water and isopropanol after each measurement. The background used was the spectrum obtained in air.

### 3.3. Procedures

Cyclic voltammetry (potential range studied was −0.4 V and 1.3 V), electrochemical impedance spectroscopy (potential range −1 V to 1 V) and FTIR method (wavelength range 4000–500 cm^−1^) were used to characterize the electrode surface. Cyclic voltammetry at the same potential range was used to evaluate the electrochemical behaviour of the modified sensors in HT solution and their detection and quantification in real samples.

The free radical galvinoxyl was used to determine the antiradical capacity of the samples by recording the absorbance variation by UV-Vis spectrophotometry.

### 3.4. SPE-SWCNT Manufacturing

The modification procedure is consistent with the literature and similar to that used in another previous work [83,84], using carbon-based screen-printed electrodes (SPE-C) as the substrate. The diameter of the working electrode was 0.4 cm, resulting in a geometric area of 0.1257 cm^2^.

The single-layer carbon nanotubes (SWCNT) suspension was prepared by adding 10 mg single-layer carbon nanotube powder to 10 mL of solvent (a mixture of dimethylformamide: water (1:1)) followed by sonication for 60 min at 59 kHz.

The drop-and-dry method was used to disperse the nanomaterial suspension on the surface of the carbon-based screen-printed (SPE-C) sensors. A total of 10 μL of the suspension was poured onto the SPE-C surface, adding 5 μL at each step using an Eppendorf micropipette, thus constructing the single-layer carbon nanotube-based screen-printed (SPE-SWCNT) sensor. Solvent evaporation was carried out at room temperature in a desiccator.

### 3.5. Tyrosinase Immobilisation

The biosensor was prepared using the SPE-SWCNT electrode as a support. A volume of 10 μL was added by the pouring technique in two successive steps, leaving a drying time of 2 h.

**Figure 14:**
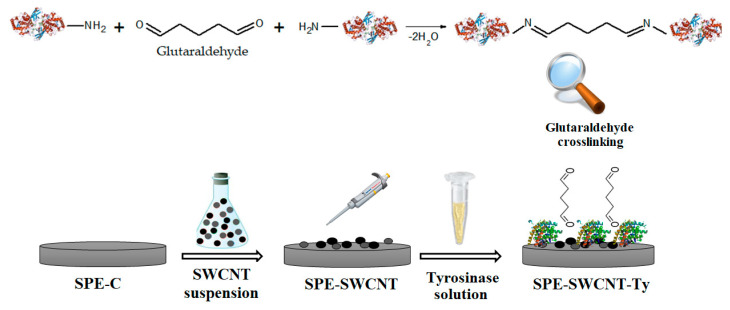
Preparation process of the tyrosinase biosensor supported on a carbon-based screen-printed electrode.

After drying, the enzyme was cross-linked by exposing the electrodes to 2% glutaraldehyde vapour for 1 min. By cross-linking, tyrosinase immobilization on the active electrode surface is ensured. Glutaraldehyde has been used in several research studies for enzyme cross-linking in various biosensitive systems [85,86]. Biosensors were stored at 4 °C until use, for a maximum of 72 h [87]. Figure 14 shows the preparation process of the tyrosinase-based biosensor supported on a previously constructed single-layer carbon nanotube-based screen-printed electrode. Glutaraldehyde binds by cross-linking with the reactive -NH_2_ groups in the structure of tyrosinase, thus ensuring the activity and stability of the enzyme.

## 4. Conclusions

The experimental study demonstrated the feasibility of the modified single-layer carbon nanotube and tyrosinase-based biosensor for the determination of hydroxytyrosol in commercial extra virgin olive oils. From the results obtained, it can be concluded that tyrosinase is a sensitive element favouring selectivity for HT detection.

The voltammetric method used in the detection but also for the study of the electrochemical behaviour of the biosensor in the chosen concentration range was cyclic voltammetry. The enzyme biosensor shows high sensitivity and selectivity for amperometric detection of HT. The concentrations of HT obtained by SPE-SWCNT-Ty in commercial extra virgin oils were close, proving their superior quality.

The newly developed biosensor based on single-layer carbon nanotubes and tyrosinase has multiple advantages, such as sensitivity, selectivity, feasibility and low cost. SPE-SWCNT-Ty could also be used in routine analyses for quality control of food products such as vegetable oils. However, further studies are needed to validate the results with a reference method (spectrophotometric, spectrometric or chromatographic).

Furthermore, future research could focus on the development of miniaturised and portable arrays combined in the same device sensors for colour, taste and odour analysis or even enzymatic biosensors capable of selectively, accurately and quickly determining the different phenolic compounds present in EVOO. Another research perspective would be the development of a lab-on-a-chip device useful in the routine analysis of olive oil or at different stages of production, from harvesting to commercialisation.

## Figures and Tables

**Table 1:** The values of the parameters obtained from the cyclic voltammograms of all the electrodes immersed in 10^−4^ M HT solution (the electrolyte support was 10^−1^ M PBS of pH 7.0).

Electrode	*E*_pa_ ^1^ (V)	*E*_pc_ ^2^ (V)	*E*_1/2_ ^3^ (V)	*I*_pa_ ^4^ (µA)	*I*_pc_ ^5^ (µA)	*I*_pc_/*I*_pa_
SPE−SWCNT	0.0856	−0.123	0.018	4.682	−6.725	1.436
SPE−SWCNT−Ty	0.1004	−0.003	0.048	30.419	−46.096	1.515

^1^ Potential of the anodic peak; ^2^ potential of the cathodic peak; ^3^ half-wave potential; ^4^ Current of the anodic peak; ^5^ Current of the cathodic peak.

**Table 2:** Linear equation (*I*_pc_ vs. v), R^2^ and Γ for the two electrodes used in the analysis.

Electrode	Linear Equation	R^2^	Γ (mol × cm^−2^)
SWCT-SPE	*I*_pc_ = −20.116 × 10^−6^ v − 5.2438 × 10^−6^	0.9941	3.903 × 10^−11^
SPE-SWCNT-Ty	*I*_pc_ = −238.77 × 10^−6^ v − 27.653 × 10^−6^	0.9951	4.618 × 10^−10^

**Table 3:** Linear dependence equation, R^2^, LOD and LOQ for the two modified electrodes.

Electrode	Linear Equation	R^2^	LOD (M)	LOQ (M)
SPE-SWCNT-Ty	y = −0.341x − 22.245	0.9762	3.49 × 10^−8^	1.0 × 10^−7^

**Table 4:** Characteristic parameters of SPE-SWCNT-Ty for HT.

Analyte	SPE-SWCNT-Ty		
	*I*_max_/μA	*h*	$K_{M}^{app}/\mu M$
HT	−24.271	0.817	0.0723

**Table 5:** Interference of chemically related compounds on the quantitative determination of HT 10^−4^ M.

Interfering Compound	Ratio	Recovery/%
Oleuropein	1:1	100 ± 3.4
Tyrosol	1:1	98 ± 2.5

**Table 6:** Concentrations of HT in commercial EVOO obtained by the voltammetric method.

No	Commercial EVOO	mg/kg HT (±RSD) Obtained by Voltammetric Method
1	Minerva Greek Extra Virgin Olive Oil	91.4 ± 1.4
2	Terra Delyssa Huile D”Olive De Tunisie	68.7 ± 0.6
3	Monini Classico Olio Extra Vergine Di Oliva	62.1 ± 1.0
4	Costa D’Oro L’extra olive oil	110.3 ± 1.8
5	Olitalia Extra Virgin olive	107.6 ± 1.4
6	Greek Koroneiki Extra Virgin Olive Oil	33.8 ± 0.8
7	Extra Virgin Olive Oil Mazza	76.2 ± 0.9
8	Extra Virgin Olive Oil Oliol	75.1 ± 0.5
9	Pietro Coricelli Extra Vergine di Oliva Non filtrato	74.6 ± 1.1
10	Ulei de masline extravirgin Costa d’Oro Il Grezzo	89.6 ± 0.6
11	Pietro Coricelli Olio Extra Vergine di Oliva	148.0 ± 0.7
12	Extra virgin olive oil Monastir	89.2 ± 1.5

RSD—relative standard deviation.

**Table 7:** Determination of % RSC of HT in EVOO samples.

#	Sample	% RSC
1	Minerva Greek Extra Virgin Olive Oil	21.76
2	Terra Delyssa Huile D”Olive De Tunisie	13.73
3	Monini Classico Olio Extra Vergine Di Oliva	15.69
4	Costa D’Oro L’extra olive oil	21.57
5	Olitalia Extra Virgin olive	23.73
6	Greek Koroneiki Extra Virgin Olive Oil	11.76
7	Extra Virgin Olive Oil Mazza	15.73
8	Extra Virgin Olive Oil Oliol	15.76
9	Pietro Coricelli Extra Vergine di Oliva Non filtrato	15.53
10	Ulei de masline extravirgin Costa d’Oro Il Grezzo	17.69
11	Pietro Coricelli Olio Extra Vergine di Oliva	30.89
12	Extra virgin olive oil Monastir	18.65

## Data Availability

Not applicable.
